# Development and Optimization of Sodium Deoxycholate-Stabilised Shellac-Based Polymeric Nanoparticles for Enhanced Pharmacokinetics and Anticancer Activity of Etodolac in Hepatocellular Carcinoma

**DOI:** 10.3390/polym18151863

**Published:** 2026-07-29

**Authors:** Ritesh A. Fule, Aishwarya S. Rangurwar, Pankaj D. Nagpure, Helal G. Alanazi, Mohammed M. Alruwaili, Md Ali Mujtaba, Gamal Osman Elhassan, Siham Abdoun

**Affiliations:** 1 Department of Pharmaceutical Science, Dadasaheb Balpande College of Pharmacy, Rashtrasant Tukadoji Maharaj Nagpur University, Nagpur 440037, Maharashtra, India; 2 Department of Medical Laboratory, College of Applied Medical Sciences, Prince Sattam bin Abdulaziz University, Al-Kharj 11942, Saudi Arabia; 3 Department of Medical Laboratory Technology, College of Applied Medical Sciences, Northern Border University, Arar 73213, Saudi Arabia; 4 Center for Health Research, Northern Border University, Arar 73213, Saudi Arabia; 5 Department of Pharmaceutics, College of Pharmacy, Qassim University, Buraidah 51452, Saudi Arabia

**Keywords:** etodolac, hepatocellular carcinoma, nanoparticles, shellac, bioavailability, cytotoxicity

## Abstract

Background/Objectives: Hepatocellular carcinoma, or liver cancer, is the sixth most common cancer worldwide and the third leading cause of cancer-related mortality. Therefore, this study aimed to develop and optimise etodolac-loaded sodium deoxycholate-stabilised shellac-based polymeric nanoparticles (ETD-SDS-SHNPs) to improve the pharmacokinetic profile of etodolac (ETD) in HepG2 human hepatocellular carcinoma (HCC) cells. Methods: The nanoprecipitation method was used to prepare ETD-SDS-SHNPs, which were then optimised using a Box–Behnken design and evaluated for particle size (PS), zeta potential (ZP), entrapment efficiency (EE), solid-state characterization, and in vitro drug dissolution. Furthermore, in vivo oral bioavailability, cytotoxicity, cellular uptake, and cell cycle progression were assessed. Results: The optimised ETD-SDS-SHNPs formulation showed an average PS of 192 ± 1.50 nm, ZP of −20.2 ± 0.4 mV, and EE of 81.6 ± 1.42%. Differential scanning calorimetry and X-ray diffraction confirmed the reduced crystallinity of ETD-SDS-SHNPs, indicating partial amorphisation of the drug in the formulation. Field-emission scanning electron microscopy revealed predominantly spherical nanoparticles with relatively smooth surfaces. The peak plasma concentration (C_max_) of ETD-SDS-SHNPs (11.86 ± 0.12 µg/mL) was significantly higher than that of the pure ETD (7.15 ± 0.14 µg/mL). Similarly, the AUC_0–t_ for ETD-SDS-SHNPs (351.63 ± 4.59 µg·h/mL) was approximately 1.48-fold greater than that of the pure drug (236.09 ± 4.60 µg·h/mL), indicating enhanced bioavailability. ETD-SDS-SHNPs demonstrated dose-dependent enhancement of cytotoxicity against HepG2 cells, accompanied by S-phase cell cycle arrest and increased cellular uptake. Conclusions: ETD-SDS-SHNPs formulation offers an enhanced passive, efficient, and safer nanocarrier platform for repurposing etodolac in liver cancer therapy, showing significant promise for improving clinical outcomes in HCC treatment.

## 1. Introduction

The rapidly rising costs of new cancer treatments are likely to be beyond the means of many countries’ health systems. Repurposing the wide variety of already approved non-cancer medications is an alluring substitute that might offer cancer patients better treatment alternatives. Significant advantages of this strategy include reduced costs, faster development, and safer preclinical and clinical testing procedures [1]. One promising approach to increasing productivity is to repurpose existing medications for novel therapeutic applications, thereby shifting a large portion of the development effort to biotechnology companies. As the number of successful cases in this field continues to grow, more businesses are now exploring repositioning opportunities within the current pool of approved medications [2].

Hepatocellular carcinoma (HCC), also referred to as liver cancer, is one of the deadliest types of cancer and the fifth most common type in humans. An estimated one million people may be impacted by liver cancer by 2025, making the increasing number of cases a serious global health concern. Worldwide, it is the third leading cause of cancer-related deaths and the sixth most common cancer overall [3]. Recent data suggested that, in addition to viral hepatitis and liver disease brought on by alcohol consumption, metabolic syndrome and non-alcoholic liver disease may be important contributors to the development of HCC [4]. Furthermore, research indicates that within two years after a hepatectomy, more than 80% of patients experience recurrent tumours in the remaining liver tissue [5]. Disruption of apoptosis and uncontrolled cell proliferation are considered key factors contributing to the development and progression of hepatocellular carcinoma (HCC), as resistance to apoptosis limits the effectiveness of standard anticancer therapies [6,7]. The suppression of programmed cell death, also known as apoptosis, is a crucial factor in cancer development [8]. This disruption of apoptotic pathways may significantly contribute to treatment resistance and help prevent cell death, uncontrolled cell growth, and the regrowth of many tumours [9,10].

Etodolac (ETD) is a nonsteroidal anti-inflammatory drug (NSAID) approved by the U.S. Food and Drug Administration (FDA) and acts as a selective cyclooxygenase-2 (COX-2) inhibitor. In addition to relieving postoperative pain, ETD is widely used to treat osteoarthritis, rheumatoid arthritis, and ankylosing spondylitis [11,12]. ETD exerts its anticancer activity through multiple mechanisms, including inhibition of cyclooxygenase-2 (COX-2), modulation of peroxisome proliferator-activated receptor gamma (PPARγ), suppression of nuclear factor kappa-B (NF-κB) signaling, and downregulation of cyclin D1, thereby inhibiting tumour cell proliferation and inducing apoptosis [13,14]. Previous studies have demonstrated the anticancer potential of etodolac against several malignancies, including prostate and colorectal cancers, where it inhibited cancer cell proliferation and exhibited promising anticancer activity. These findings support the repurposing of etodolac as a potential anticancer agent [13]. However, despite these promising anticancer properties, the clinical application of ETD in hepatocellular carcinoma remains limited because of its poor aqueous solubility, low oral bioavailability, rapid systemic elimination, and insufficient tumour accumulation. Several delivery systems have been investigated to improve the pharmaceutical performance of etodolac, including nanoemulsions and lipid-based nanocarriers. These systems have demonstrated improved drug solubility, sustained release, and enhanced bioavailability. However, they still exhibit limitations such as poor physical stability, limited drug loading, and insufficient tumor-specific drug delivery. Therefore, in the present study, sodium deoxycholate-stabilized shellac-based polymeric nanoparticles were developed to overcome these limitations and improve the pharmacokinetic profile and anticancer efficacy of etodolac in hepatocellular carcinoma [15,16].

The FDA has classified shellac (SH) as Generally Recognized as Safe (GRAS), making it a safe and suitable material for nanoparticle preparation. SH is a naturally occurring resin extracted from the secretions of lac insects and is composed primarily of aleuritic acid together with cyclic terpene acids, including jalaric acid, shellolic acid, laccijalaric acid, and laccishellolic acid, which collectively form the polymeric resin matrix of shellac [17]. It is a naturally biodegradable oligoester that has been widely used to enhance bioavailability [18]. SH nanoparticles (SHNPs) have demonstrated controlled drug delivery through multiple routes of administration [19,20]. SH possesses high drug-binding capacity, excellent biocompatibility, biodegradability, low toxicity, and efficient drug delivery capability, making it an attractive polymer for nanoparticle formulation [21,22]. Several substances, including sodium caseinate, dextran sulfate, and sodium deoxycholate, have been proposed as coating agents for shellac nanoparticles (SHNPs) to improve their colloidal stability [23,24]. Compared with these stabilizers, sodium deoxycholate has been shown to effectively stabilize SHNPs [25]. Shellac was selected because of its FDA-recognized GRAS status, excellent biocompatibility, biodegradability, pH-responsive sustained drug release, and high drug-loading capacity. Sodium deoxycholate was selected as an effective stabilizer and surfactant because it enhances nanoparticle stability, drug encapsulation efficiency, wettability, cellular membrane permeability, and intestinal absorption, thereby improving oral bioavailability.

The objective of this study was to develop and optimize sodium deoxycholate-stabilized shellac nanoparticles to improve the oral bioavailability and anticancer efficacy of repurposed etodolac against hepatocellular carcinoma. Furthermore, the optimized formulation was evaluated for its physicochemical properties, in vitro drug release, cytotoxicity, cell-cycle analysis, cellular uptake, and in vivo pharmacokinetics to establish its potential as a nanocarrier system for liver cancer therapy. We hypothesized that encapsulating etodolac within sodium deoxycholate-stabilized shellac nanoparticles would improve its aqueous solubility, oral bioavailability, cellular uptake, and anticancer efficacy by enhancing sustained drug release and intracellular delivery.

## 2. Materials and Methods

### 2.1. Materials

Etodolac was purchased from Biochemical Products, Mumbai, India, whereas shellac was obtained from Research Lab Fine Chem Industries, Mumbai, India. Sodium deoxycholate, ethanol, and methanol were procured from HiMedia Laboratories Pvt. Ltd., Mumbai, India. Mannitol was obtained from Sisco Research & Lab Pvt. Ltd., Mumbai, India. All chemicals and solvents used were of analytical grade.

### 2.2. Cell Lines and Cell Culture

The human hepatocellular carcinoma cell line (HepG2) was cultured in RPMI-1640 medium (Gibco, Grand Island, NY, USA) supplemented with 10% fetal bovine serum (Gibco, Grand Island, NY, USA), 100 µg/mL streptomycin, and 100 U/mL penicillin. The cells were maintained at 37 °C in a humidified incubator with 5% CO_2_.

### 2.3. Methods

#### 2.3.1. Formulation of Etodolac-Loaded Sodium Deoxycholate Stabilised Shellac-Based Polymeric Nanoparticles (ETD-SDS-SHNPs)

ETD-SDS-SHNPs were prepared using the nanoprecipitation method [26]. Nine preliminary formulation batches were prepared by varying the ETD:SH ratio and the sodium deoxycholate concentration. The corresponding amounts of ETD and SH (1:3 ratio) were dissolved in 10 mL of 85% ethanol, and the mixture was vortexed for 5 min. The organic phase was then subjected to probe sonication (SONICS Vibra Cell VC750, Newtown, CT USA) at a frequency of 20 kHz for 30 min. The resulting ethanolic solution was added dropwise using a syringe into deionised water containing sodium deoxycholate under continuous magnetic stirring at 450 rpm. The mixture was stirred for 3 h at room temperature to ensure complete evaporation of ethanol. The final dispersion was centrifuged at 11,180× *g*. Mannitol (5% *w*/*v*) was added as a cryoprotectant, and the nanoparticle dispersion was frozen at −80 °C overnight prior to lyophilization. Freeze-drying was performed using a Christ Alpha 1–2 LDplus lyophilizer (Martin Christ Gefriertrocknungsanlagen GmbH, Osterode am Harz, Germany). Primary drying was carried out at −50 °C under a vacuum pressure of <0.05 mbar for a total lyophilization time of 48 h to obtain dry nanoparticles.

#### 2.3.2. Formulation Optimization Using BBD

The formulation was systematically optimized using the BBD with Design-Expert software version 13 (Stat-Ease Inc., Minneapolis, MN, USA). The amount of SH (X_1_), the ultra-probe sonication time (X_2_), and the sodium deoxycholate (X_3_) are the three most important factors. These were chosen as independent variables (factors) for optimisation at three levels: low (−1), medium (0), and high (+), as indicated in Table 1. As shown in Table 2, the BBD comprised 17 experimental runs with five replicated center points. For the formulation of polymeric nanoparticles, the following parameters were examined as dependent responses: particle size (PS) (Y_1_), entrapment efficiency (EE) (Y_2_), and drug release (DR) (Y_3_). This study examined the effects of three independent variables on various formulation characteristics. Every variable was evaluated to determine its contribution to formulation optimisation. A 3^3^ BBD with three dependent and independent variables was used to optimise the level of variables in the ETD-SDS-SHNPs formulation. The data was entered into BBD, and the results were analysed through mathematical modelling. One-way analysis of variance (ANOVA) was used to analyse the data-fitting component of the quadratic second-order model, along with other parameters such as the predicted residual sum of squares, the predicted R^2^, and the coefficient of determination R^2^. The numerical desirability function and graphical optimisation techniques were used to determine the optimal conditions for the formulation of polymeric nanoparticles [27].

### 2.4. Characterization of ETD-SDS-SHNPs

All physicochemical characterization experiments (particle size, zeta potential, entrapment efficiency, drug loading, dissolution, and stability studies) were performed in triplicate (*n* = 3).

#### 2.4.1. Particle Size (PS), Polydispersity Index (PDI), and Zeta Potential (ZP) Determination

The PS, PDI and ZP of the ETD-SDS-SHNPs formulations were determined using a particle size analyzer (Anton-Paar Litesizer 500, GmbH, Graz, Austria). To prevent aggregation, the samples were diluted with deionised water and sonicated. The average PS (Z-average) and PDI were measured at 25 °C. A low PDI (<0.5) suggests a uniform, stable nanoparticle, whereas a PS value indicates nanoscale suitability for drug delivery [28]. The ZP of the samples was determined using the same instrument. ZP is a measure of the colloidal stability of nanoparticles and reflects their surface charge. Particle aggregation is inhibited by strong electrostatic repulsion and preserves the stability of the nanoparticle dispersion, as indicated by a high absolute value [29].

#### 2.4.2. %Entrapment Efficiency (%EE) Determination

A sample of ETD-SDS-SHNPs formulation (10 mg) was solubilized in ethanol. After that, the sample was centrifuged in centrifugation machine (REMI Elektrotechnik Ltd., Mumbai, India) for 15 min at 11,180× *g*. The supernatant was then filtered, diluted with ethanol, and analysed using UV spectroscopy at λ_max_ 278 nm to determine the concentration of unencapsulated ETD. The concentration of free drug was calculated using a calibration curve prepared from standard etodolac solutions in ethanol over a concentration range of 2–20 µg/mL, which showed excellent linearity (R^2^ > 0.999). The %EE was determined using the following formula [30].

(1) $$\%EE=\frac{Amount\;of\;drug\;added-Amount\;of\;Free\;drug\;in\;supernatant}{Amount\;of\;drug\;added}\times 100$$

#### 2.4.3. Drug Loading (DL) Determination

DL of ETD-SDS-SHNPs was determined using a solvent extraction and indirect quantification approach. Accurately weighed nanoparticles (10 mg) were dissolved in ethanol to disrupt the nanoparticle matrix and release the entrapped drug. Although the nanoparticles were completely dissolved, the solution was centrifuged at 11,180× *g* for 15 min to remove any trace amounts of insoluble shellac residues and other particulate impurities, if present. The clear supernatant was then filtered to eliminate any remaining particulate impurities that could interfere with UV–visible spectrophotometric analysis, suitably diluted with ethanol, and the drug content was quantified at λmax 278 nm against a validated calibration curve. The %DL was calculated using the following formula [30].

(2) $$\%DL=\frac{Amount\;of\;drug\;in\;nanoparticle}{Total\;weight\;of\;nanoparticles}\times 100$$

### 2.5. Solid-State Characterization of ETD-SDS-SHNPs

#### 2.5.1. Fourier-Transform Infrared Spectroscopy (FTIR) Analysis

The samples ETD, sodium deoxycholate, SH, physical mixture, and optimised formulation ETD-SDS-SHNPs were analysed using a Shimadzu FTIR spectrometer (Shimadzu Corporation, Kyoto, Japan). The samples were prepared using the KBr disc method, and scanning was performed between 400 and 4000 cm^−1^ [31].

#### 2.5.2. Differential Scanning Calorimetry (DSC) Analysis

The thermal behaviour and phase transition of ETD and optimised formulation ETD-SDS-SHNPs were examined using DSC (DSC-PYRIS-1, PerkinElmer, Waltham, MA, USA). 5 mg of each sample was placed in an aluminium pan and sealed. Thermograms were obtained by scanning the sample at a constant rate of 10 °C/min across a temperature range from 50 °C to 420 °C [32].

#### 2.5.3. Powder X-Ray Diffraction (PXRD) Analysis

ETD and the optimized formulation (ETD-SDS-SHNPs) were subjected to PXRD analysis using an X-ray diffractometer (Bruker D8 Advance, Madison, WI, USA). An X’celerator detector with Cu Kα radiation and a nickel-filtered graphite monochromator was used to analyze 45 kV and 40 mA. Samples were scanned at a rate of 1°/min between 3° and 45° (2θ). The PXRD data were acquired and analyzed using Diffrac. EVA Suite version V4.2.1. (Bruker AXS, Madison, WI, USA) [33].

#### 2.5.4. Field Emission Scanning Electron Microscopy (FE-SEM) Analysis

The size, shape, and surface morphology of ETD-SDS-SHNPs were examined using FE-SEM (JEOL JSM-6390LV, Tokyo, Japan). The lyophilized nanoparticles were mounted on aluminum stubs using conductive carbon tape and sputter-coated with a thin gold layer (approximately 8–10 nm) to minimize charging effects and improve image quality. The samples were then observed under vacuum at an accelerating voltage of 15 kV [34].

### 2.6. In Vitro Drug Dissolution Study and Release Kinetics

A dialysis membrane (molecular weight cut-off: 12 kDa) was used for the in vitro drug release study. The dialysis membrane was pre-soaked in distilled water for 12 h before use. Sink conditions were maintained throughout the dissolution study by replacing the withdrawn sample volume with an equal volume of fresh release medium. The pre-soaked dialysis bag was filled with an ETD suspension or ETD-SDS-SHNPs (equivalent to 10 mg of ETD), securely tied, and immersed in 250 mL of phosphate-buffered saline (PBS, pH 7.4) containing 1% (*v*/*v*) Tween 80. The system was maintained at 37 ± 0.5 °C under continuous magnetic stirring at 100 rpm throughout the experiment. Aliquots (2 mL) were withdrawn at predetermined intervals (0.5, 2, 4, 6, 8, 10, 12, 24, and 48 h) and replaced with an equal volume of fresh PBS to maintain sink conditions. The release data were fitted to zero-order, first-order, Higuchi, and Korsmeyer–Peppas kinetic models to determine the drug release mechanism [15,35]. The study was performed in triplicate (*n* = 3).

### 2.7. Stability Studies

For storage stability, the optimized formulation was stored at 40 ± 2 °C/75 ± 5% RH. Samples were analyzed on Day 0, Day 7, Day 14, and Day 30 for particle size (PS), entrapment efficiency (EE), drug loading (DL), and in vitro drug release (DR) [31]. All experiments were performed in triplicate (*n* = 3).

### 2.8. In Vivo Oral Bioavailability Study

#### 2.8.1. Animals

The Institutional Animal Ethics Committee (IAEC) of Dadasaheb Balpande College of Pharmacy, Besa, Nagpur, India, authorised the in vivo experimental protocol under protocol number DBCOP/IAEC/1426/2024-25/P-19 with date of approval 2 May 2025. The ARRIVE guidelines were followed in conducting the study.

#### 2.8.2. Pharmacokinetic Study

The oral bioavailability and pharmacokinetics of the optimized ETD-SDS-SHNPs formulation were assessed in male Sprague–Dawley (SD) rats (200–250 g). The rats were kept in conventional housing with free access to food and water before the experiment and were acclimated to a temperature of 22 ± 2 °C for 1 week. They were fasted overnight before dosing. A total of 15 rats were randomly divided into three groups (five animals per group): Group I was the control group (*n* = 5), and Group II (*n* = 5) received a suspension of free ETD in normal saline. In contrast, Group III (*n* = 5) was administered the optimized ETD-SDS-SHNPs formulation in normal saline. Both groups received an oral gavage dose of ETD at 30 mg/kg based on body weight. Heparinised microcentrifuge tubes were used to collect blood samples at predetermined intervals. Plasma was separated by centrifugation at 6000 rpm for 15 min at 4 °C and then kept at −20 °C for further analysis. Samples of plasma were thawed to room temperature, processed using a one-step protein precipitation method, and analyzed by validated RP-HPLC [36].

PK Solver software version 2.0 was used to determine pharmacokinetic parameters through the non-compartmental analysis method. Drug concentrations in blood plasma and a variety of pharmacokinetic parameters were evaluated separately. The mean ± standard deviation (SD) was used to express the results. ANOVA was used to test for statistical significance at the 5% level. At *p* < 0.05, differences were deemed statistically significant [37].

### 2.9. In Vitro Anticancer Cell Line Studies

#### 2.9.1. Cytotoxicity Assay

The cytotoxicity of ETD and its nanoparticle formulation (ETD-SDS-SHNPs) was assessed in HepG2 liver cancer cells using the MTT assay. HepG2 cells (10,000 cells/well) were seeded into 96-well plates and cultured at 37 °C in a humidified atmosphere containing 5% CO_2_ using RPMI-1640 medium supplemented with 10% fetal bovine serum (FBS) and 1% antibiotics. After 24 h of incubation, the cells were treated with different concentrations of ETD or ETD-SDS-SHNPs prepared in serum-free medium. The final DMSO concentration was maintained below 1%. After 24 h of treatment, MTT solution (0.5 mg/mL) was added to each well, and the plates were incubated for 4 h. The resulting formazan crystals were dissolved in DMSO, and the absorbance was measured at 570 nm using a microplate reader. IC_50_ values were calculated using GraphPad Prism 6 and expressed as mean ± SD. Microscopic analysis was performed using an Olympus inverted microscope equipped with an AmScope camera [38,39,40,41,42]. All experiments were performed in triplicate (*n* = 3).

#### 2.9.2. Cell Cycle Analysis by Flow Cytometry

Flow cytometry was employed to evaluate the effect of ETD and ETD-SDS-SHNPs on cell-cycle progression in HepG2 liver cancer cells. Cells were seeded and treated with ETD or ETD-SDS-SHNPs at their respective IC_50_-equivalent concentrations for 24 h, while untreated cells served as the control group. Following incubation, the cells were trypsinized, transferred into 1.5 mL microcentrifuge tubes, and washed once with 500 µL PBS. Approximately 1 × 10^6^ cells were resuspended in 100 µL PBS and gently vortexed to obtain a homogeneous single-cell suspension. The cell suspension was fixed by adding 900 µL of 70% ethanol and incubated for at least 2 h at 4 °C. The fixed cells were then centrifuged, and the pellet was resuspended in 500 µL of propidium iodide (PI) staining solution containing 0.1% Triton X-100, 10 µg/mL PI, and 100 µg/mL DNase-free RNase A in PBS. The mixture was incubated in the dark for 30 min at room temperature before analysis using a flow cytometer. Cell-cycle distribution was analyzed using FCS Alyzer software version 0.9.13-alpha. The fluorescence intensity of PI was detected using the FL-2 channel, with an excitation wavelength of 488 nm and an emission wavelength of approximately 617 nm [43].

#### 2.9.3. Cellular Uptake by Confocal Microscopy

HepG2 cells were obtained from NCCS, Pune, India. To prepare the slides, ultraclean coverslips were placed into six-well plates, with one coverslip per well. 200 µL of APTES (an aminosilane coupling agent used to improve glass surface hydrophilicity and enhance cell adhesion during confocal imaging) was added to each well to ensure the coverslips were completely covered. After 1 h of incubation, the APTES solution was returned to the stock, and the wells were washed twice with complete medium to remove residual APTES. Cells were planted at a density of 5000–10,000 cells/well after being collected at 90% confluency. The FITC stock concentration (1 mg/mL in DMSO) was prepared. Following a 24 h incubation at 37 °C with 5% CO_2_, the plates were transferred to fresh culture medium containing ETD-SDS-SHNPs at an ETD-equivalent concentration of 10 µg/mL together with FITC (1 µL/mL) and incubated for a further 24 h.

Following incubation, the cells were fixed for 20 min at room temperature using 1 mL of 3% paraformaldehyde after the culture media was withdrawn. Following a 30 min incubation period with 100 µL of DAPI (1 µg/mL) for nuclear staining, the cells were washed with PBS. After that, coverslips were carefully placed on pristine glass slides. An inverted laser-scanning confocal microscope ZEISS LSM 900 (Carl Zeiss Microscopy GmbH, Jena, Germany) was used to analyse the produced samples. Three channels were used to collect images: DAPI (blue), FITC (green), and the combined channel. Fluorescence intensity and intracellular localization were analyzed using ImageJ version 1.54d [44,45].

### 2.10. Statistical Analysis

All in vitro experiments were performed in triplicate (*n* = 3), whereas the in vivo pharmacokinetic study was conducted using five animals per group (*n* = 5). Data are expressed as mean ± standard deviation (SD). Statistical analysis was performed using GraphPad Prism version 6.0 (GraphPad Software, San Diego, CA, USA). One-way analysis of variance (ANOVA) was used for comparison between groups, and differences were considered statistically significant at *p* < 0.05.

## 3. Results and Discussion

### 3.1. Formulation Optimization of ETD-SDS-SHNPs

This study investigated the repurposing potential of ETD, a COX-2 Inhibitor NSAID, for the treatment of HCC by formulating it into sodium deoxycholate-stabilised shellac nanoparticles (ETD-SDS-SHNPs). The formulation aimed to overcome ETD’s poor solubility and to enhance its anticancer activity. ETD-SDS-SHNPs formulation optimization was statistically performed by using the BBD. A set of 17 experiments was developed using BBD to optimize the formulation, with three independent and three dependent variables (Table 1). ETD-SDS-SHNPs were prepared to achieve an optimum PS, %EE, and % drug release. The amounts of SH, Probe sonication time, and Sodium deoxycholate were chosen as independent variables (factors) at three distinct levels: low (−1), medium (0), and high (+). Table 2 displayed the results for each batch. A quadratic model was used to fit the acquired data, and the fitting analysis was carried out using several statistical parameters [46].

### 3.2. Factor—Response Relationship and Response Surface Mapping

#### 3.2.1. Effect of Independent Variables on PS

The effect of SH amount (A), probe sonication time (B), and sodium deoxycholate amount (C) on PS of ETD-SDS-SHNPs was evaluated using the BBD. The following polynomial equation for the PS was generated:

PS = 195 + 10A + 27.50B + 5C − 30AB − 10AC + 15BC − 37.50A^2^ + 7.50B^2^ + 27.50C^2^ (3)

**Figure 1:**
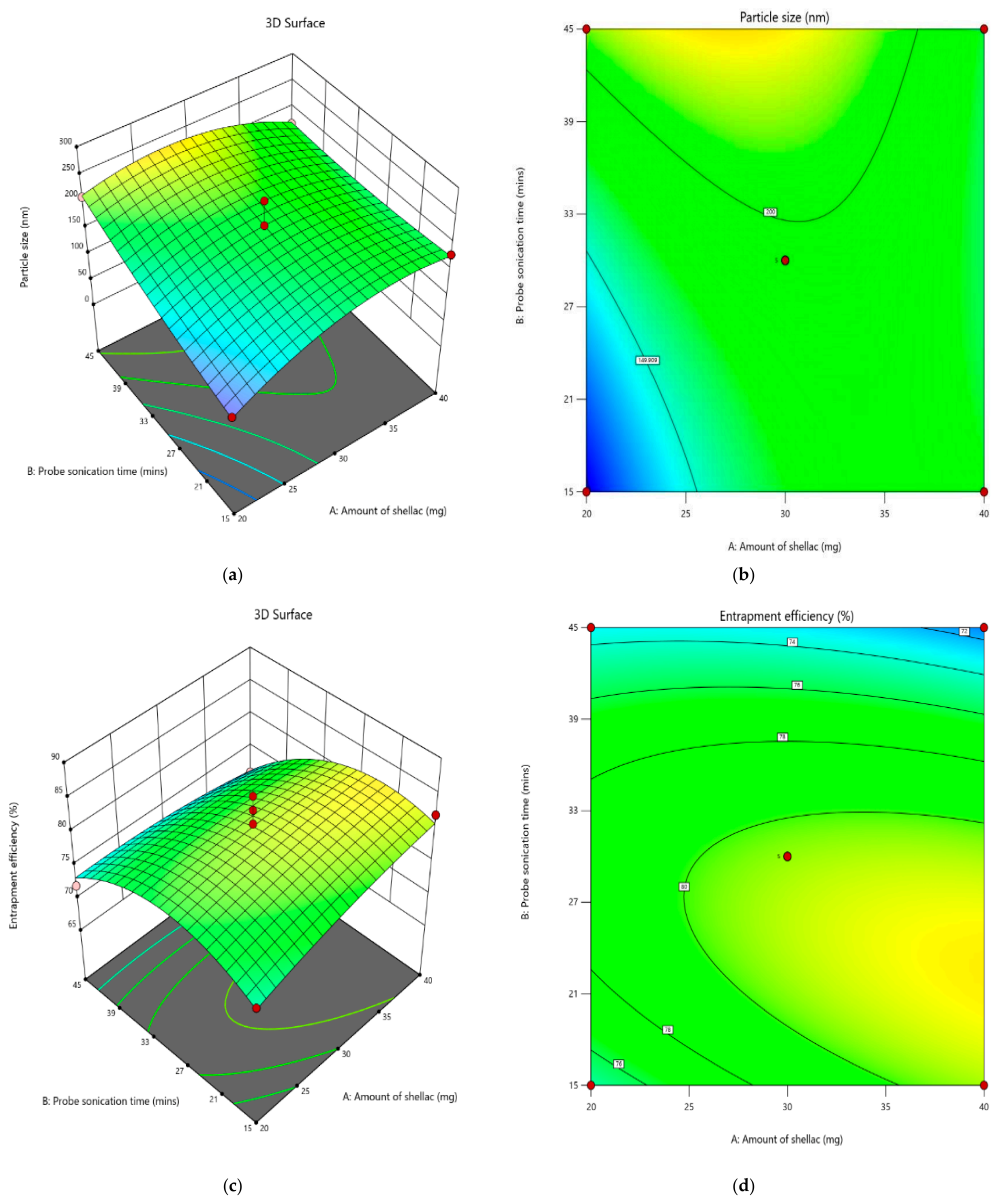

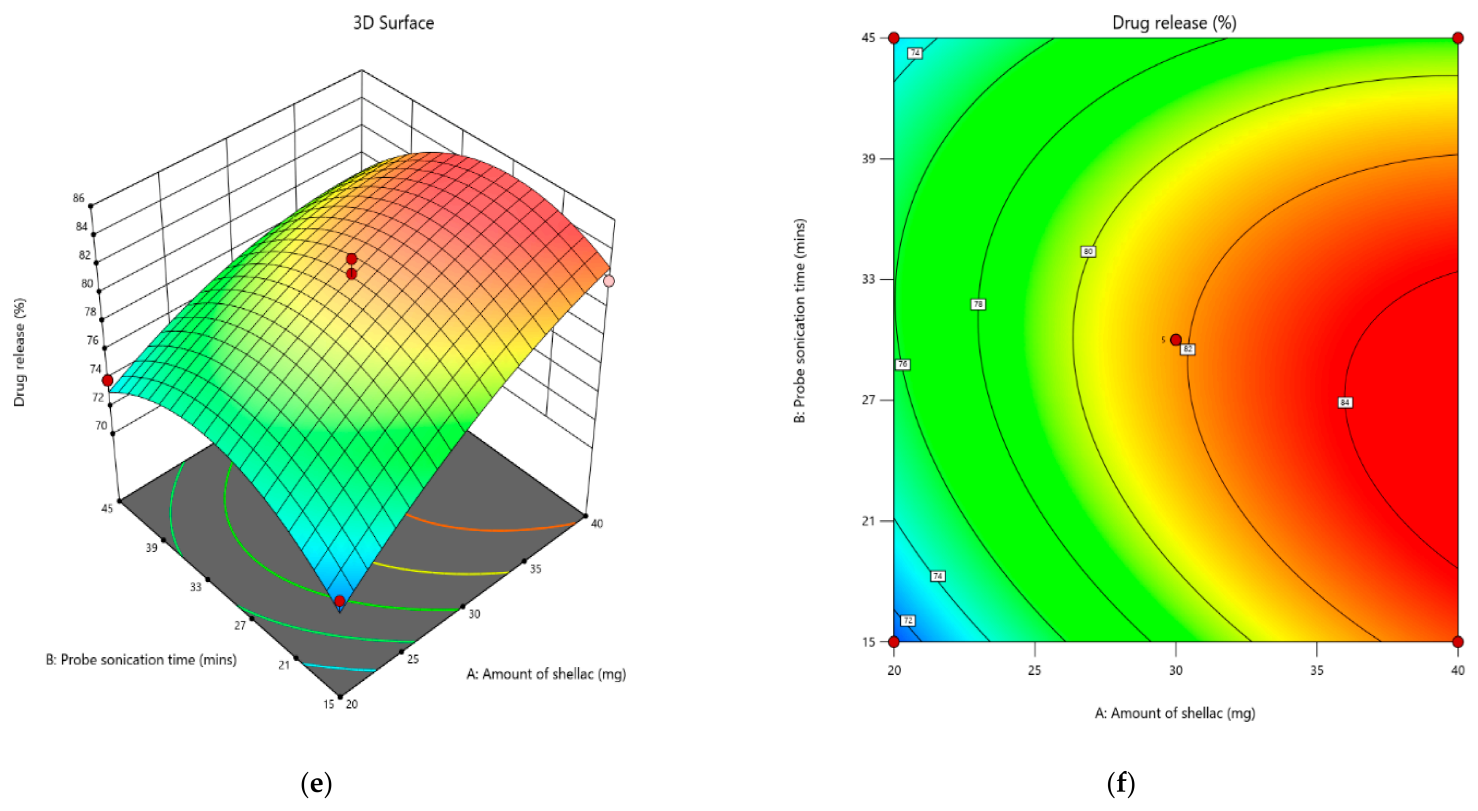
Counter plot and 3D-response surface plot showing the effect of amount of SH and probe sonication time on particle size (**a**,**b**), entrapment efficiency (**c**,**d**) and drug release (**e**,**f**).

The coded equation demonstrates how each of the three independent factors affects PS. Among the linear effects, sonication time (B) had the greatest beneficial effect, followed by SH (A) and sodium deoxycholate (C). This suggests that larger PS is caused by micelle formation or aggregation and is increased by longer sonication and higher levels of polymer or surfactant. Interestingly, the interaction terms AB and AC had negative coefficients, suggesting that SH, when combined with surfactant or sonication, improves emulsification and dispersion and reduces PS. On the other hand, the BC interaction increased, perhaps due to overprocessing or micelle swelling. While positive B^2^ and C^2^ values show that excessive sonication or surfactant levels can enlarge particles. The counterplot (Figure 1a) and 3D response surface plot (Figure 1b) show the effects of SH amount and probe sonication time on PS. For PS, the model showed an F-value of 3.90 with a *p*-value of 0.0431, indicating that the model is statistically significant (*p* < 0.05). The lack-of-fit *p*-value (*p* = 0.7967) was not significant, confirming that the model fits the data adequately. Overall, the model emphasises that to effectively control PS, all three variables must be precisely optimised [47].

#### 3.2.2. Effect of Independent Variables on %EE

The effects of SH amount (A), probe sonication time (B), and sodium deoxycholate amount (C) on %EE were assessed using the BBD. The quadratic model for %EE is as follows:

%EE = 80.60 + 1.00A − 2.75B + 0.25C − 2.00AB − 4.50AC − 4.50BC − 0.80A^2^ − 4.80B^2^ + 0.70C^2^ (4)

The BBD demonstrated how SH (A), probe sonication time (B), and sodium deoxycholate (C) affected %EE. While sonication time had a significant negative impact, most likely because of drug leakage from excessive energy input, SH demonstrated a slight positive effect, improving entrapment through matrix formation. A slight positive effect was observed with sodium deoxycholate, which may have enhanced emulsification. %EE was adversely affected by the interaction terms (AB, AC, and BC), suggesting that high combinations of these parameters may destabilize the system. The quadratic terms, B^2^ had the most negative impact, A^2^ had a slight decrease, and C^2^ had a slight increase. To achieve the maximum %EE in nanoparticle formulations, the model highlights the need to precisely optimise polymer concentration, sonication time, and surfactant concentration. The counterplot (Figure 1c) and 3D response surface plot (Figure 1d) show the effect of SH amount and probe sonication time on %EE. In the case of %EE, the quadratic model was also statistically significant, with an F-value of 3.76 and a *p*-value of 0.0472. Furthermore, the non-significant lack-of-fit value (*p* = 0.7158) confirms the suitability of the model for describing the observed data [48].

#### 3.2.3. Effect of Independent Variables on Drug Release

The effects of SH amount (A), sonication time (B), and sodium deoxycholate amount (C) on drug release were assessed using the BBD. The quadratic model was found to be as follows:

Drug release = 81.80 + 4.38A − 0.50B + 2.13C − 1.50AB − 1.25AC − 1.00BC − 1.53A^2^ − 3.78B^2^ − 2.02C^2^ (5)

SH had the greatest linear effect, suggesting that higher polymer levels may improve matrix swelling and enhance drug diffusion. While sonication time had a slight negative impact, perhaps because of the formation of dense particles that impede diffusion, sodium deoxycholate also had a positive effect on release, most likely by improving emulsification and permeability. Combinations of higher variable levels may decrease drug release, possibly by increasing matrix compactness, as indicated by the negative interaction terms (AB, AC, and BC). Additionally, quadratic terms indicated that high concentrations of each component, especially sonication time (B), decreased release, highlighting the need for rational optimisation. All things considered, optimising drug release from nanoparticles requires balanced amounts of polymer, surfactant, and sonication. The counterplot (Figure 1e) and 3D response surface plot (Figure 1f) show the effects of SH amount and sonication time on drug release. For drug release, the quadratic model exhibited a higher F-value (16.93) and a highly significant *p*-value (0.0006), indicating a strong influence of the independent variables on the response. Moreover, the non-significant lack-of-fit (*p* = 0.3602) further confirms the model’s adequacy and reliability [49].

### 3.3. Optimization and Validation of ETD-SDS-SHNPs

**Figure 2:**
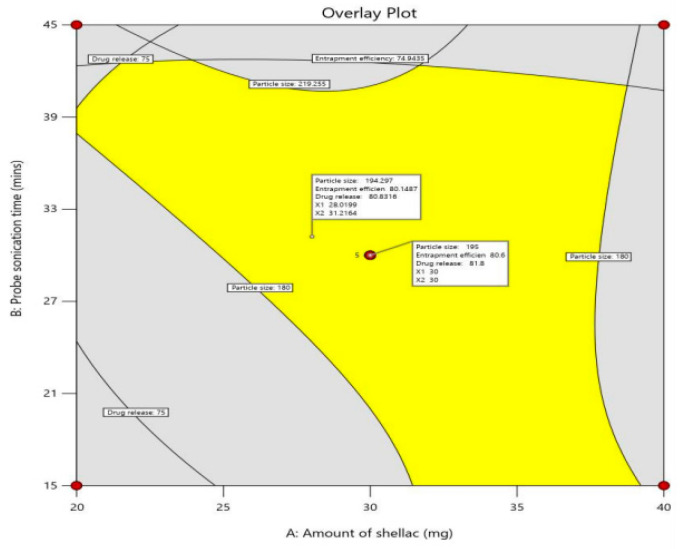
Overlay plot of response variables of batches of ETD-SDS-SHNPs1 and ETD-SDS-SHNPs2.

The combined effects of SH amount (A) and sonication time (B) on PS, %EE, and drug release are shown in the Box–Behnken overlay plot, with sodium deoxycholate (C) fixed at 200 mg (Figure 2). The ideal design space, where all response parameters satisfy the required standards (minimum PS, maximum EE, and drug release), is shown by the yellow area. Grey areas, on the other hand, indicate less-than-ideal conditions, either due to reduced entrapment and increased PS at higher A and B levels, or to low drug release (<75%) at lower A and B levels. Overall, the findings demonstrate that balanced SH levels and sonication time are crucial for achieving optimal formulation performance. Subsequently, two checkpoint formulations (ETD-SDS-SHNPs1 and ETD-SDS-SHNPs2) were prepared and assessed for PS, %EE, and DR. The experimental and projected response values were compared to validate the BBD outcomes. The data in Table 3 indicate that the BBD optimization technique was significantly effective in optimizing the formulation of ETD-SDS-SHNPs. The anticipated error was within 5%, and the experimental and predicted values of the best formulation exhibited no significant difference (*t*-test, *p* > 0.05) [50]. A well-balanced formulation appropriate for nanoparticle drug delivery is indicated by the highlighted optimal point (30 mg SH, 30 min sonication), which yields 80.6% EE, 81.8% drug release, and a PS of 195 nm.

### 3.4. Characterization of Optimized ETD-SDS-SHNPs Formulation

#### PS, PDI, ZP, %EE and %DL Determination

**Figure 3:**
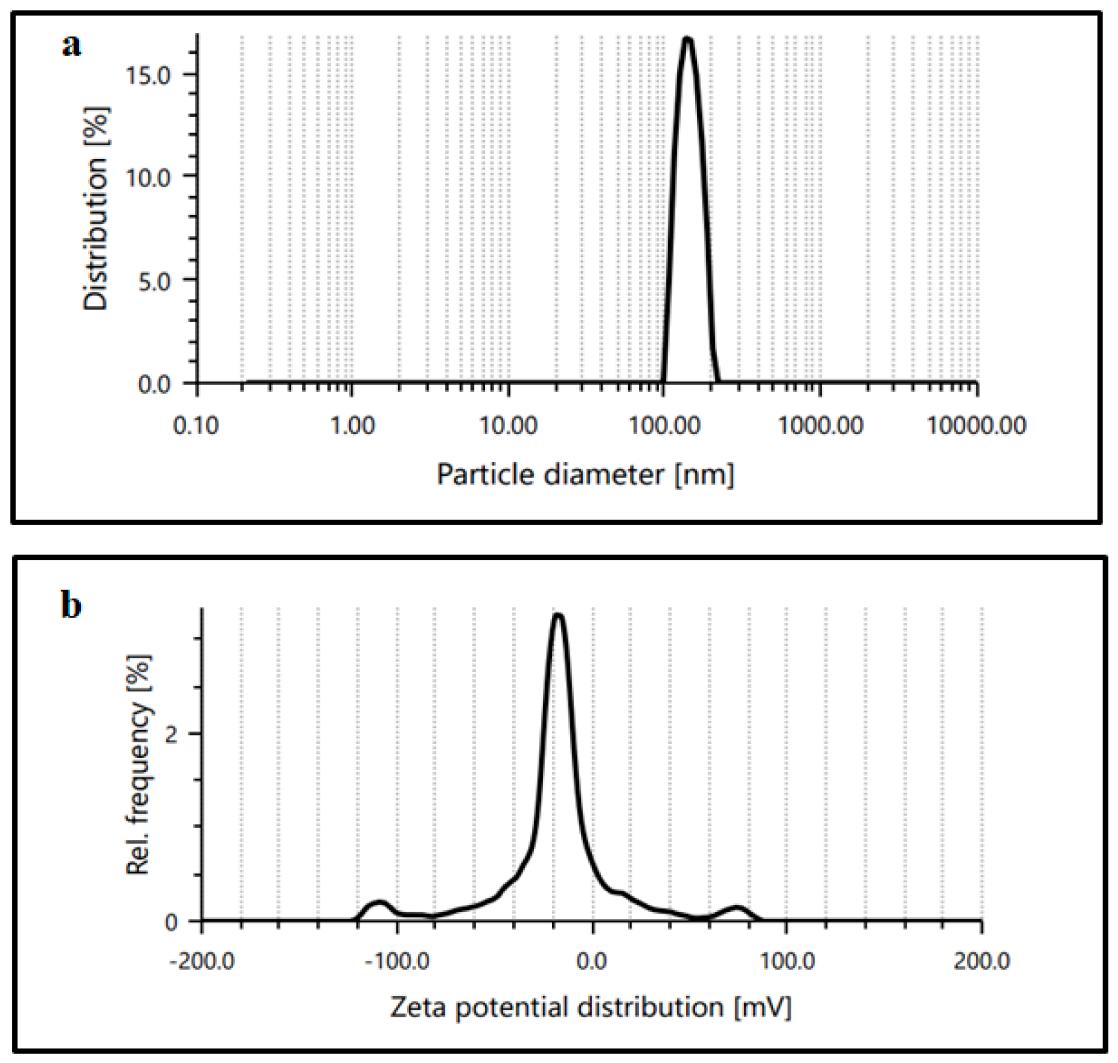
Particle size distribution (**a**) and zeta potential (**b**) of the optimized ETD-loaded sodium deoxycholate-stabilized shellac-based polymeric nanoparticles (ETD-SDS-SHNPs).

The PS of the optimized ETD-SDS-SHNPs was 192 ± 1.50 nm (Figure 3a). For oral drug delivery applications, the observed PS fell within an ideal range. With a narrow PDI range and a PDI of 0.297 ± 0.002, the results showed good dispersion stability and uniformity. The ZP of −20.2 ± 0.4 mV was obtained, confirming the nanoparticles’ stability (Figure 3b). This preserves their integrity, increases their bioavailability, and expands their potential as a drug delivery system for anticancer treatments. A significant amount of the active drug was effectively encapsulated within the polymeric nanoparticle, as evidenced by the EE of 81.6 ± 1.42% [51]. The PDI is an important parameter derived from dynamic light scattering (DLS) that reflects the width of the particle size distribution and the homogeneity of the nanoparticle population. In the present study, the optimized formulation exhibited a PDI value of approximately 0.28, indicating a relatively narrow particle size distribution. According to commonly accepted criteria for polymeric nanoparticle systems, PDI values below 0.3 are considered indicative of a monodisperse and uniform nanoparticle population, whereas values above 0.5 generally represent broad size distributions and poor formulation homogeneity. The observed PDI values, therefore, suggest that the developed ETD-SDS-SHNPs formulation possesses good colloidal uniformity and controlled particle size distribution. These findings suggest that the combined electrostatic stabilization provided by sodium deoxycholate and the polymeric shellac matrix contributes to maintaining nanoparticle dispersion stability, thereby preventing aggregation and drug leakage over time [49].

The calculated DL for the optimized formulation was found to be approximately 20.1 ± 0.84%, which indicates efficient incorporation of ETD within the shellac nanoparticle matrix. DL provides additional insight into the formulation efficiency and dosage feasibility, demonstrating that the developed nanoparticle system can deliver a therapeutically relevant amount of drug while maintaining nanoscale characteristics and acceptable EE. Furthermore, the obtained DL values are consistent with those reported for polymeric nanoparticle systems designed for hydrophobic drug delivery [50].

### 3.5. Solid-State Characterization

#### 3.5.1. Fourier-Transform Infrared Spectroscopy (FTIR) Analysis

**Figure 4:**
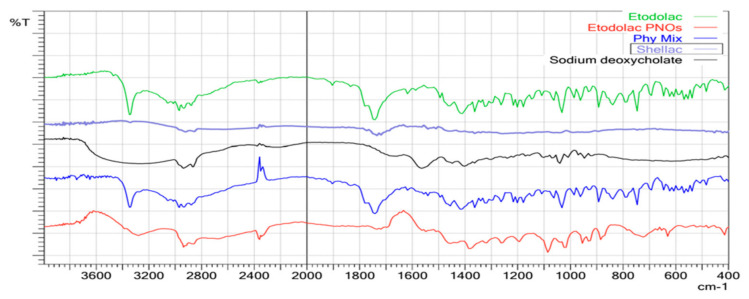
FTIR overlay spectra of pure etodolac (ETD), shellac (SH), sodium deoxycholate, ETD-loaded sodium deoxycholate-stabilized shellac-based polymeric nanoparticles (ETD-SDS-SHNPs), and the physical mixture (Phy Mix).

FTIR analysis was performed to investigate the functional group interactions between ETD and the excipients in the optimized ETD-SDS-SHNPs. The FTIR spectrum of pure ETD exhibited several prominent peaks (Figure 4). These include a broad O-H stretching around 3300–3400 cm^−1^ because of the presence of the hydroxyl group, a sharp C=O stretching vibration around 1725 cm^−1^ that is equivalent to the carboxylic acid group, an aromatic C=C stretching near 1600 cm^−1^, and an aliphatic C–H stretching between 2850 and 2950 cm^−1^. These numbers act as benchmarks for spotting interactions or structural alterations. The FTIR spectrum of the ETD-SDS-SHNPs formulation differs noticeably from that of the pure drug (Figure 4). The drug may be hydrogen-bonded or encapsulated within the polymeric matrix as the C=O stretching peak at 1725 cm^−1^ is shifted to a lower wavenumber or exhibits decreased intensity. Around 3300 cm^−1^, the O–H stretching band widens and diffuses, suggesting stronger hydrogen bonding interactions with sodium deoxycholate and SH. To further confirm interaction and successful formulation, the bending vibrations of the C-H groups also appear slightly shifted or subdued. Without much shifting or broadening, the physical mixture of the drug and excipients in Figure 4 maintains the distinct peaks of the polymers and ETD. Since the components are physically combined rather than molecularly bonded or dispersed, there is no chemical interaction. In contrast to the pure drug and physical mixture, it emphasises how distinctive peaks in the formulation spectrum vanish, shift, and widen. Peak shifting, particularly in the carbonyl and hydroxyl regions, and broadening of the O-H stretching are signs that the drug ETD was successfully incorporated into the polymeric nanoparticles. They also suggest that hydrogen bonding and encapsulation may have occurred, thereby improving the drug’s stability and compatibility within the nanoparticulate system [52].

#### 3.5.2. Differential Scanning Calorimetry (DSC) and Powder X-Ray Diffraction (PXRD) Analysis

**Figure 5:**
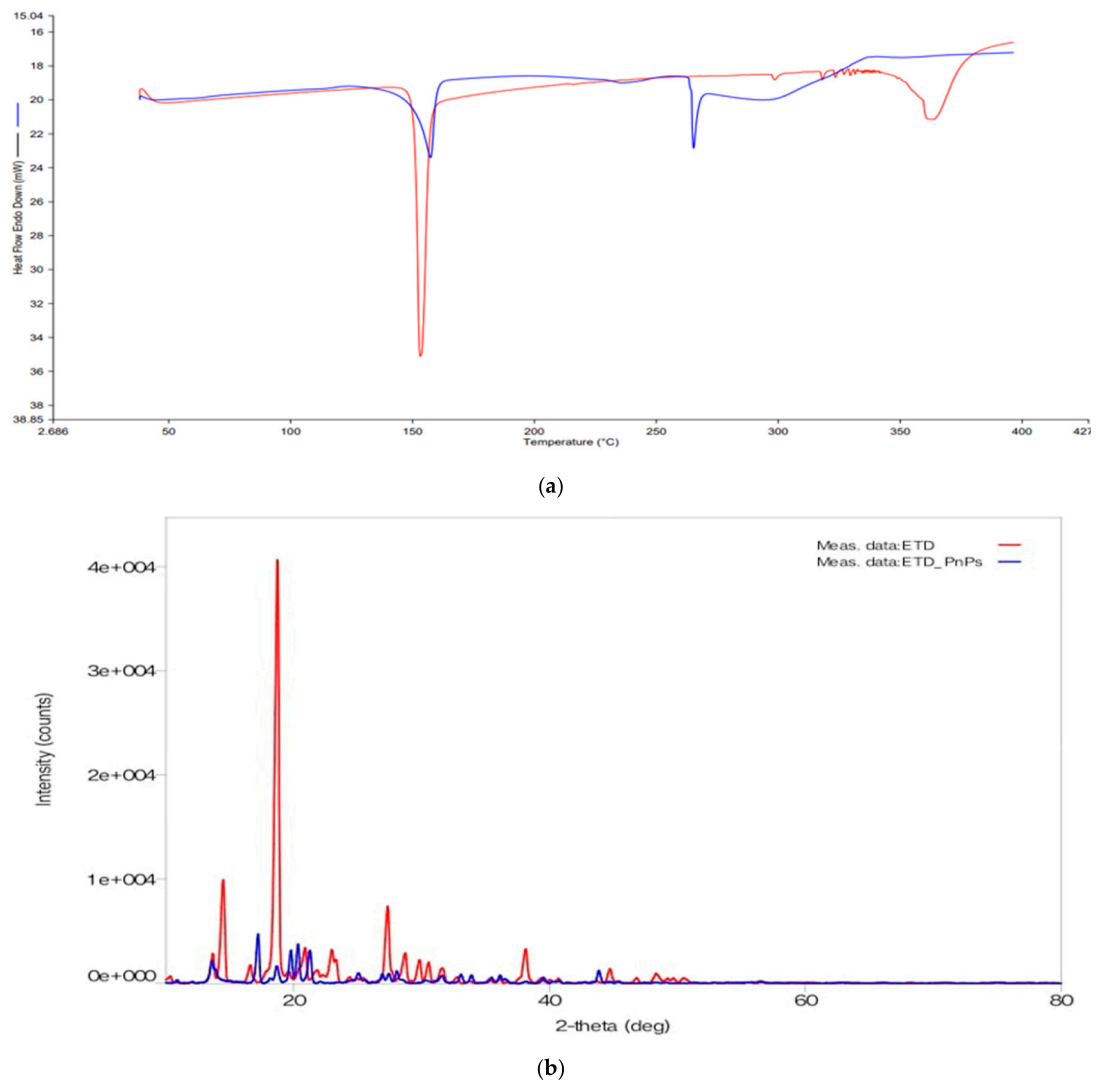
(**a**) DSC thermograms of pure ETD (red line) and ETD-SDS-SHNPs (blue line); (**b**) PXRD diffractograms of pure ETD (red line) and ETD-SDS-SHNPs (blue line).

The DSC thermogram of pure ETD shows a sharp endothermic peak at its melting point, 153 °C, indicating its crystalline nature. As opposed to this, the DSC thermogram of ETD-SDS-SHNPs displays either a broad peak or a notable decrease in the melting endotherm, suggesting that the drug has changed into an amorphous or partially crystalline form within the nanoparticle formulation (Figure 5a). This transformation is beneficial because the amorphous state is commonly associated with improved solubility and bioavailability [53].

The XRD diffractogram of the pure ETD drug shows multiple sharp and intense peaks at 14.49°, 17.29°, 19.52°, 22.27°, 24.41°, and 26.76°, indicating its crystalline nature (Figure 5b, red line). In contrast, the XRD diffractogram of the ETD-SDS-SHNPs formulation (Figure 5b, blue line) exhibited a significant reduction in both the number and intensity of diffraction peaks compared to pure ETD. Although some of the original peaks, such as those around 2θ = 18.12°, 20.90°, and 26.01°, were still detectable, they appeared broader and much weaker, indicating a considerable loss in crystallinity. Additionally, the spectrum showed a relatively broad hump at 2θ = 21.04°, characteristic of amorphous or semicrystalline structures. This suggests that nanoparticle formation disrupted ETD’s crystalline lattice, possibly due to drug entrapment within the nanoparticle matrix or interactions with the carrier/stabilizer during formulation. These findings are consistent with previous reports demonstrating that polymeric nanoparticle formulations reduce drug crystallinity and promote partial amorphization, resulting in improved drug solubility and dissolution [54].

#### 3.5.3. FE-SEM Analysis

**Figure 6:**
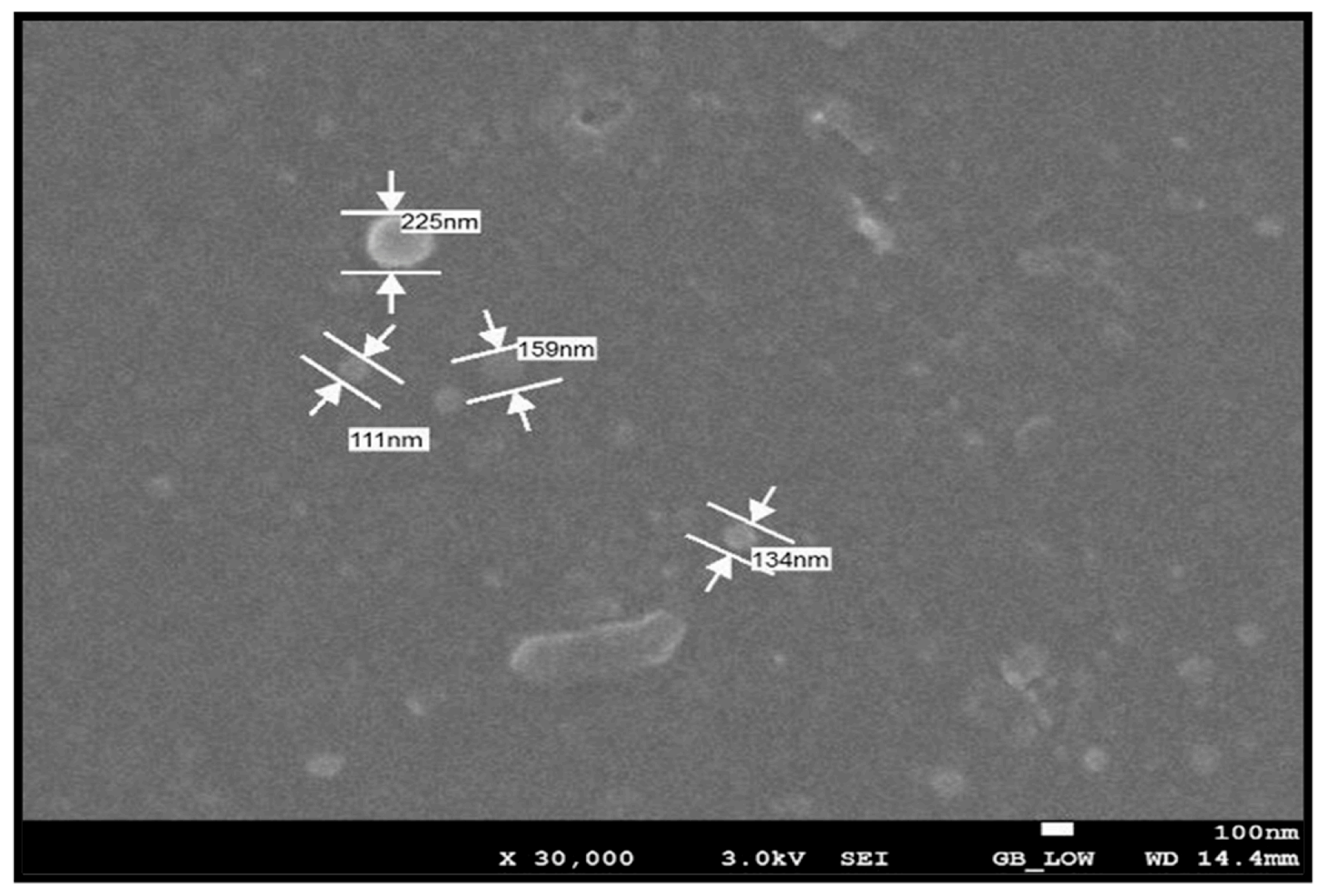
Field emission scanning electron microscopy (FE-SEM) images and particle size distribution histogram of ETD-loaded sodium deoxycholate-stabilized shellac-based polymeric nanoparticles (ETD-SDS-SHNPs). FE-SEM images were acquired at 30,000 x magnification.

The particle size and surface morphology of the optimized ETD-SDS-SHNPs formulation were examined using field emission scanning electron microscopy (FE-SEM). As shown in Figure 6, the nanoparticles were uniformly distributed, predominantly spherical, and exhibited relatively smooth surface morphology, with only a few slightly irregular particles. The particle size distribution was determined by measuring more than 100 nanoparticles using ImageJ software 1.49v. The particle size obtained from FE-SEM analysis was in good agreement with that determined by dynamic light scattering (DLS). The predominantly spherical morphology and absence of noticeable aggregation indicate the successful formation and good stability of the ETD-SDS-SHNPs formulation [55].

### 3.6. In Vitro Drug Dissolution Study and Release Kinetics

**Figure 7:**
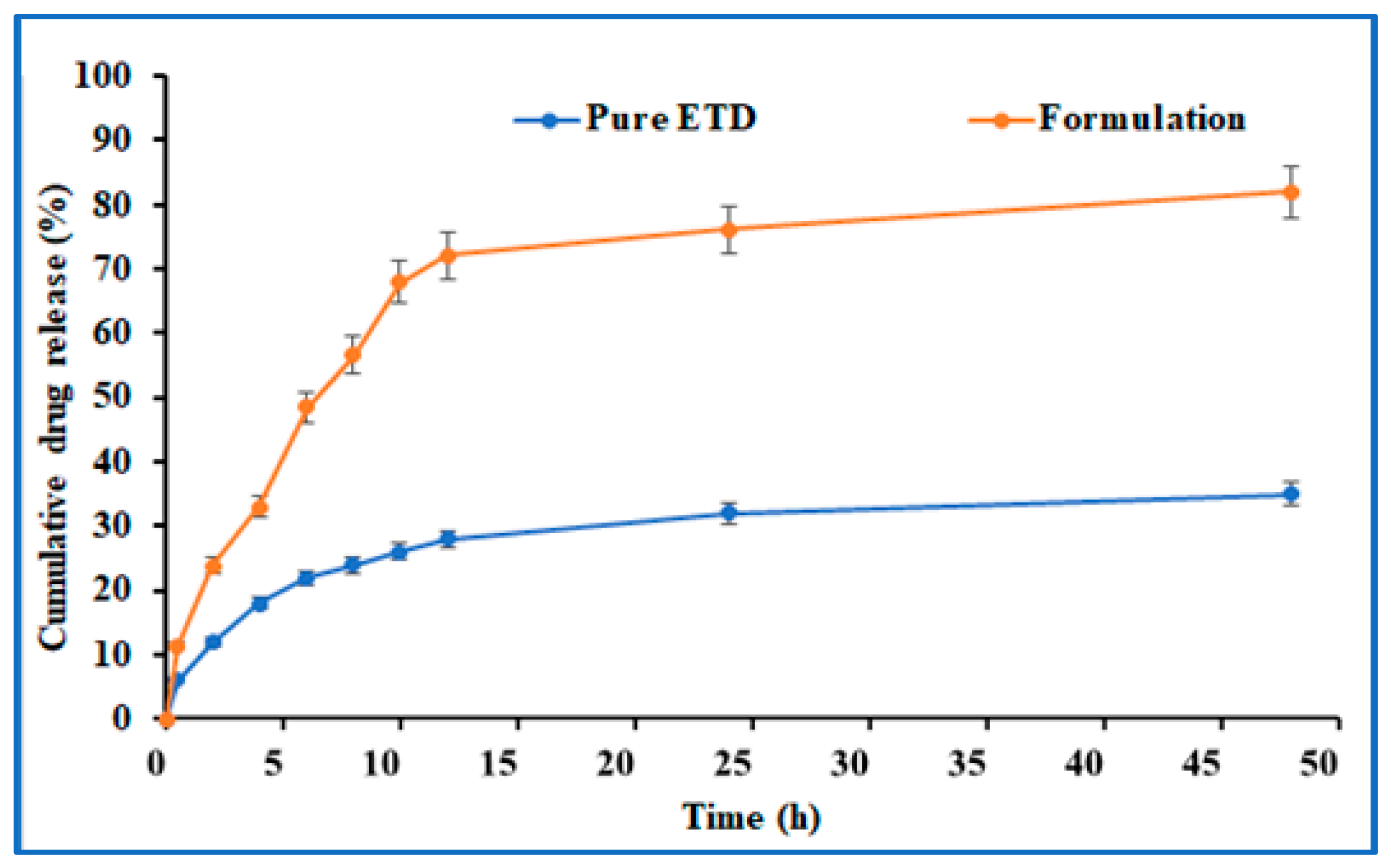
In vitro cumulative drug release profiles of pure etodolac (ETD) and the optimized ETD-loaded sodium deoxycholate-stabilized shellac-based polymeric nanoparticles (ETD-SDS-SHNPs, F17).

Achieving prolonged and sustained drug release is the primary goal of the developed ETD-SDS-SHNPs formulation, ensuring that the drug remains in systemic circulation for an extended period. Thus, it is crucial to assess its release profile. The in vitro dissolution study of pure ETD and the developed formulation in PBS at pH 7.4 is shown in Figure 7. Pure ETD exhibited limited dissolution in dissolution media, with cumulative release of around 35% even after 48 h, which can be attributed to its poor aqueous solubility and hydrophobic nature. In contrast, formulation F17 showed markedly higher and faster drug release compared to the pure drug within the same time frame [56]. The formulation releases more than 20% of the drug in the first two hours, demonstrating the initial burst release from the ETD-SDS-SHNPs formulation. At 12 h, more than 60% of the drug was released from the formulation, demonstrating rapid and effective diffusion of the drug from the polymer matrix. Conversely, the pure drug shows a more limited and slower release of 28% by the end of the study period. The sustained, higher release profile of the formulation persists for up to 48 h, ultimately achieving approximately 82% cumulative drug release at pH 7.4. This improved release behaviour can be attributed to the reduced PS, increased surface area, and the solubilizing effect of sodium deoxycholate in the nanoparticle system, which collectively enhance drug wettability, dissolution, and diffusion [56]. According to these results, the ETD-SDS-SHNPs formulation provides a better, longer-lasting drug-release profile, thereby greatly increasing the drug’s bioavailability and therapeutic effectiveness.

The in vitro release study of ETD-SDS-SHNPs exhibits first-order kinetics (R^2^ = 0.9605), suggesting that drug release is concentration-dependent. Additionally, the release data were best described by the Korsmeyer–Peppas model, with a release exponent (*n*) value of 0.391 indicating that the drug release follows a Fickian diffusion mechanism (Table 4). Drug release is governed by diffusion through the polymer matrix, with some contribution from polymer erosion. The ETD-SDS-SHNPs exhibit a first-order release pattern, with a mechanism largely driven by Fickian diffusion, consistent with the diffusion-controlled release typically observed in polymer-based drug delivery platforms. Similar sustained-release behavior has been reported in earlier studies on shellac/polymeric nanoparticles, where controlled drug diffusion from the polymeric matrix contributed to prolonged drug release [57].

### 3.7. Stability Results

The results showed no significant changes in PS, EE, DL, and DR, indicating that the nanoparticle system maintained its physicochemical integrity during storage. These findings in Table 5 suggest that the combined electrostatic stabilization provided by sodium deoxycholate and the polymeric shellac matrix contributes to maintaining nanoparticle dispersion stability, thereby preventing aggregation and drug leakage over time. A marginal increase in PS from 192 nm to 195 nm was observed over 30 days, which remained within acceptable limits and indicated minimal aggregation of nanoparticles. Similarly, the EE and DL showed negligible variation, confirming that the drug remained effectively encapsulated within the nanoparticle matrix. The drug release profile also remained consistent, suggesting that the structural integrity of the formulation was preserved during storage [31]. Overall, the results indicate that the optimized nanoparticle formulation exhibits satisfactory short-term stability.

### 3.8. Pharmacokinetics and Oral Bioavailability

**Figure 8:**
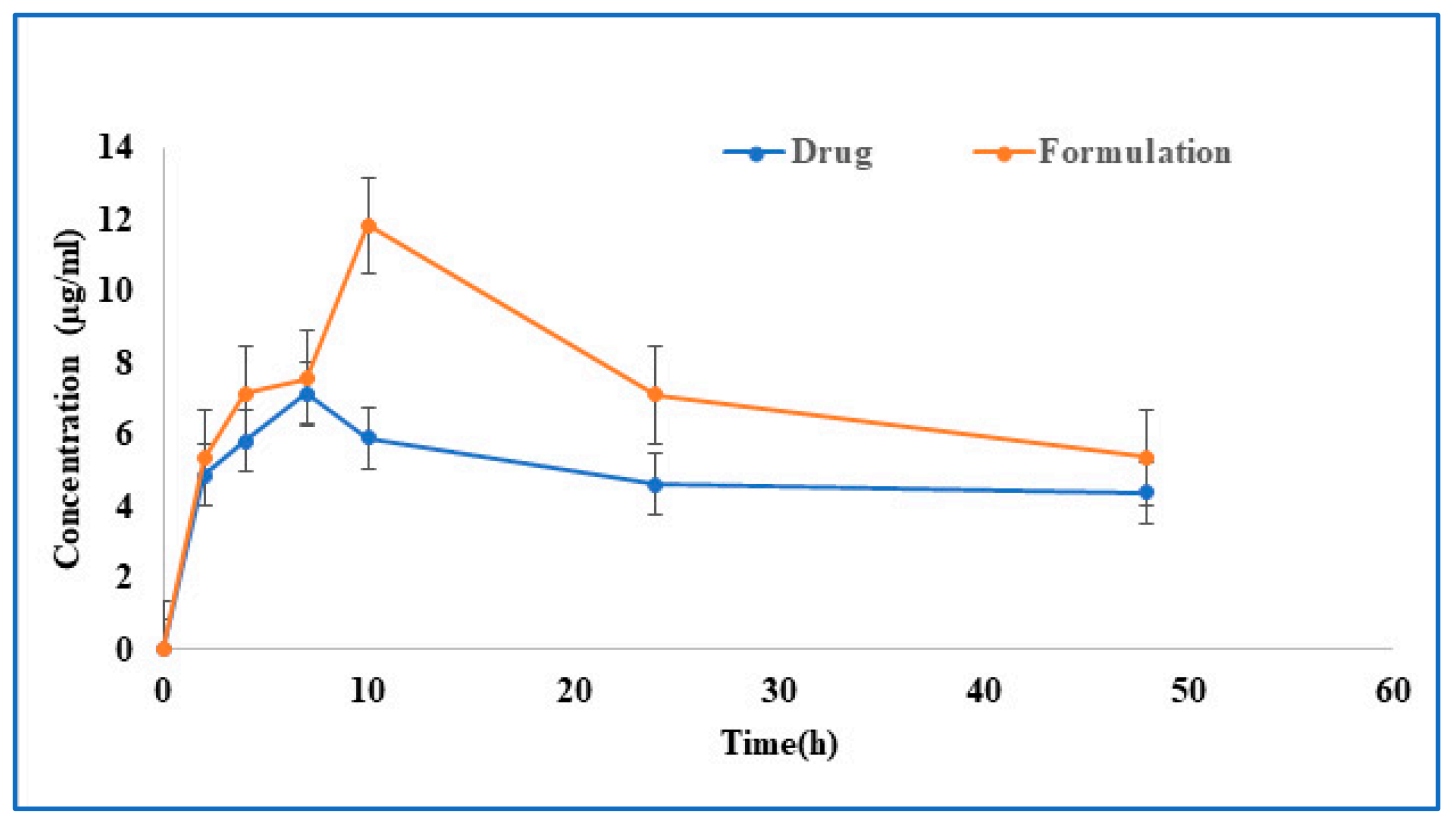
Plasma concentration–time profiles of pure etodolac (ETD) and the optimized ETD-loaded sodium deoxycholate-stabilized shellac-based polymeric nanoparticles (ETD-SDS-SHNPs, F17) following oral administration.

The pharmacokinetic behaviour of ETD was evaluated after oral administration of the optimised ETD-SDS-SHNPs formulation in Sprague–Dawley rats and compared with that of a pure ETD suspension. Pharmacokinetic parameters were calculated using non-compartmental analysis, and plasma ETD concentrations were determined using a validated RP-HPLC method. The plasma concentration–time profiles are shown in Figure 8, and the pharmacokinetic parameters are summarized in Table 6. Compared with the pure ETD suspension, the ETD-SDS-SHNPs formulation exhibited a significantly improved pharmacokinetic profile. The peak plasma concentration (C_max_) for the pure ETD and ETD-SDS-SHNPs was 7.15 ± 0.14 µg/mL and 11.86 ± 0.12 µg/mL, respectively. The area under the curve (AUC_0–t_) for ETD-SDS-SHNPs (351.63 ± 4.59 µg·h/mL) was approximately 1.48-fold higher than that of the pure drug (236.09 ± 4.60 µg·h/mL), confirming enhanced bioavailability [58]. These results suggest that the ETD-SDS-SHNPs formulation significantly improved the solubility, stability, and absorption of ETD. The enhanced oral bioavailability may be attributed to reduced PS, increased surface area, the solubilising effect of sodium deoxycholate, and the protective and controlled-release properties of SH [59].

The pharmacokinetic profile of the enhanced ETD-SDS-SHNPs formulation exhibited significant improvement over the standard ETD suspension, signifying effective alteration of drug disposal properties. The notable elevation in C_max_ (*p* < 0.001) indicates augmented systemic absorption, likely due to enhanced solubilization and permeability provided by the lipid–surfactant hybrid nanocarrier system. A significant increase in AUC_0–t_ (*p* < 0.001) further substantiates a considerable improvement in total bioavailability, with a relative bioavailability of about 149%, indicating effective drug administration and less pre-systemic loss. The T_max_ of the nanoparticle formulation was significantly extended (*p* < 0.05), indicating a delayed absorption phase compatible with sustained or controlled release behavior. This transition indicates that the SHNPs system proficiently regulates drug release kinetics, therefore sustaining therapeutic plasma concentrations for an extended duration. Conversely, the MRT exhibited no statistically significant difference (*p* > 0.05), indicating that although the rate and extent of absorption were modified, the overall residence time remained the same [58,59]. Overall, the findings confirm that ETD-SDS-SHNPs are a promising oral nanocarrier system capable of improving the pharmacokinetic performance and therapeutic efficacy of ETD in HCC therapy.

### 3.9. In Vitro Anticancer Cell Line Studies

#### 3.9.1. In Vitro Cellular Cytotoxicity

**Figure 9:**
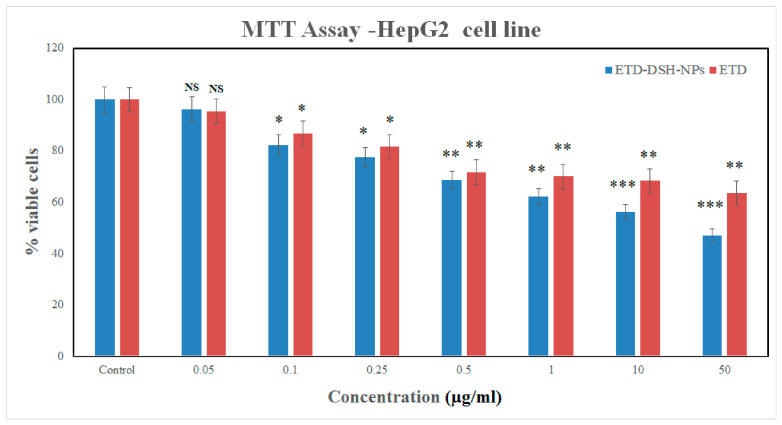
Percentage cell viability of Etodolac (pure) and optimized ETD-SDS-SHNPs in HepG2 cells. Results are expressed as percentage mean ± SD (*n* = 3) (* = *p* < 0.05 when compared with control; ** = *p* < 0.005 when compared with control, *** = *p* < 0.001 when compared with control; NS = not significant).

The cytotoxic effects of ETD and ETD-SDS-SHNPs (concentration range: 0–50 µg/mL) on HepG2 cells were assessed by MTT assay (Figure 9). Both treatments showed dose-dependent cytotoxicity; cell viability decreased with increasing concentration. ETD-SDS-SHNPs indicate higher cytotoxicity compared to free ETD. For instance, viability is decreased by ETD-SDS-SHNPs to 62.07% from 70.11% for ETD at 1 µg/mL and to 47.11% from 63.58% for ETD-SDS-SHNPs at 50 µg/mL. This implies that the formulation of ETD-SDS-SHNPs enhances anticancer activity, most likely through improved cellular uptake, sustained drug release, and increased bioavailability. This suggests that ETD-SDS-SHNPs exhibit greater cytotoxicity against HepG2 cells than pure ETD in a dose-dependent manner and have the potential to be a more effective HCC treatment option. Comparable improvements in cytotoxicity have also been reported for polymeric nanoparticle formulations owing to enhanced intracellular drug delivery and sustained drug release [60].

#### 3.9.2. Cell Cycle Analysis with Flow Cytometry

**Figure 10:**
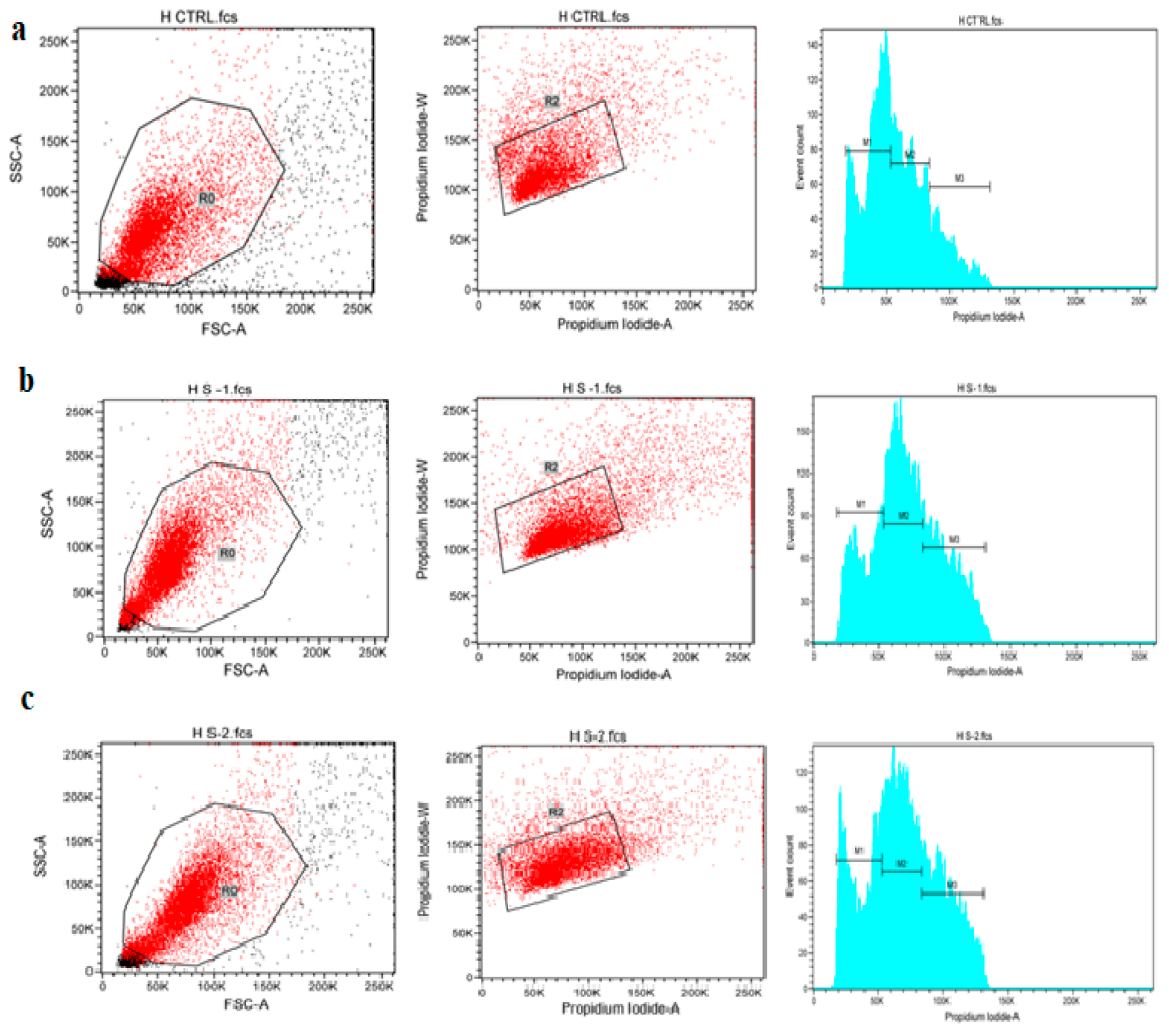

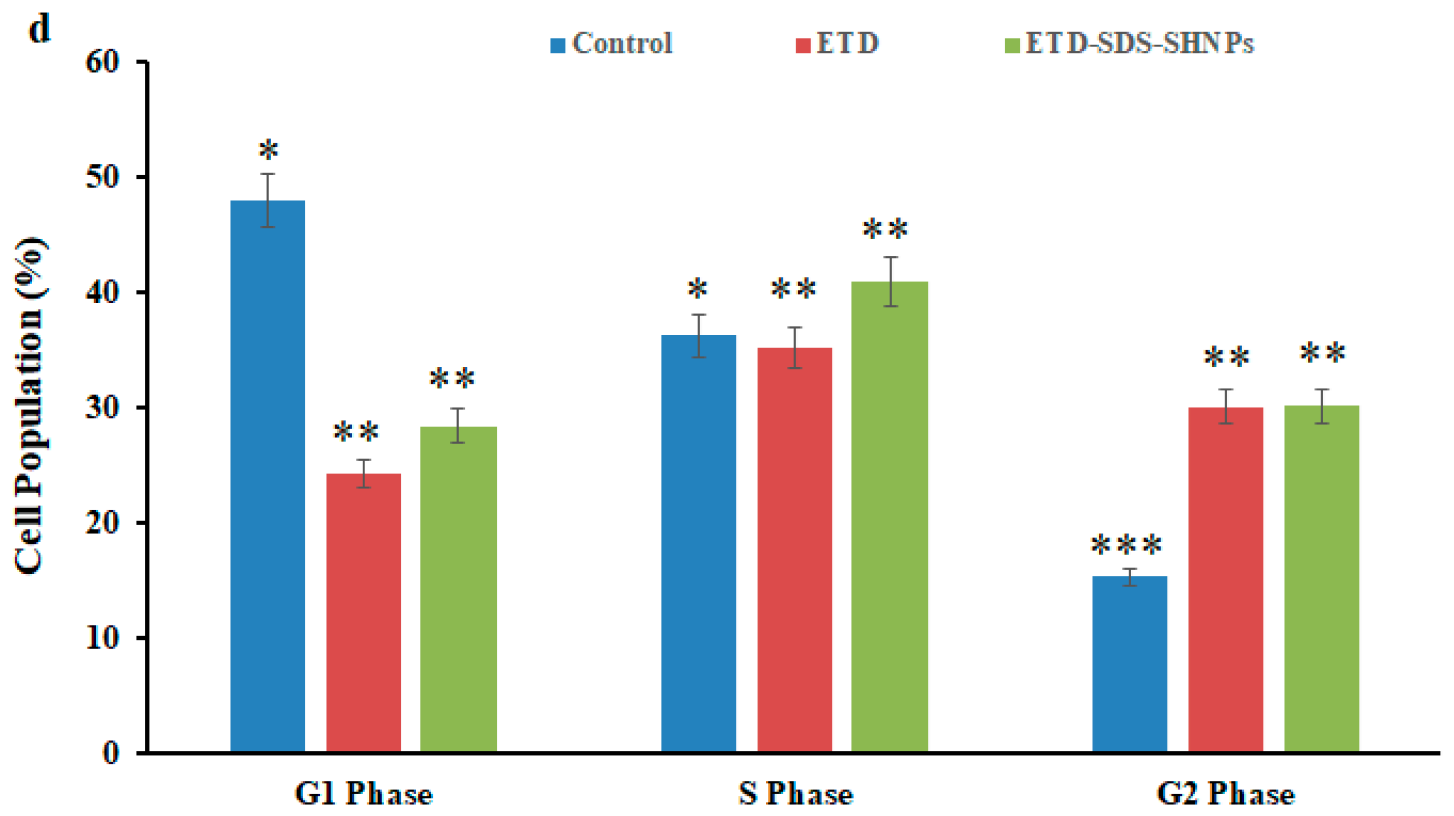
(**a**) Control group, (**b**) ETD, (**c**) ETD-SDS-SHNPs, and (**d**) graphical representation of cycle analysis with flow Cytometry on HepG2 cells. (* = *p* < 0.05 when compared with control; ** = *p* < 0.005 when compared with control, *** = *p* < 0.001 when compared with control).

Flow cytometry was performed on untreated HepG2 cells (control group), the drug-treated group, and the formulation-treated group (ETD-SDS-SHNPs), as shown in Figure 10a–c. The cell population is gated within region R0 based on internal complexity and cell size in the top-left dot plot, which displays side scatter area (SSC-A) relative to forward scatter area (FSC-A). This determines which cell population is viable for additional examination. By eliminating cell doublets or aggregates, region R2 is used to isolate single cells in the bottom left plot (Propidium Iodide-A vs. PI-W). Three regions—M1 (G1 phase), M2 (S phase), and M3 (G2/M phase) represent distinct stages of the cell cycle in the histogram of DNA content based on propidium iodide staining that is shown on the right. The sample’s cell cycle distribution changed significantly as a result of the treatment. Cell cycle progression is indicated by the highest G1 phase population (48.01%) in the Control group, which sharply declines with Drug-ETD (24.26%) and ETD-SDS-SHNPs (28.45%). Particularly with ETD-SDS-SHNPs (40.96%), S-phase accumulation rises, indicating a stop in DNA synthesis. Additionally, the G2 phase shows higher populations in treated groups (~30%) compared to the control (15.36%), suggesting inhibition of mitotic entry (Figure 10d). In general, ETD-SDS-SHNPs show improved S- and G2-phase arrest, indicating potent antiproliferative and possibly anticancer properties. Cell accumulation in the S phase following therapy with ETD-SDS-SHNPs points to a significant cell cycle arrest, suggesting that the formulation disrupts DNA synthesis. This highlights its potential as an anticancer therapy, as it contributes to cell-cycle arrest and antiproliferative effects. These findings are consistent with previous reports showing that nanoparticle-mediated drug delivery can induce cell-cycle arrest and inhibit cancer cell proliferation [61].

#### 3.9.3. Cellular Uptake Study Using Confocal Microscopy

**Figure 11:**
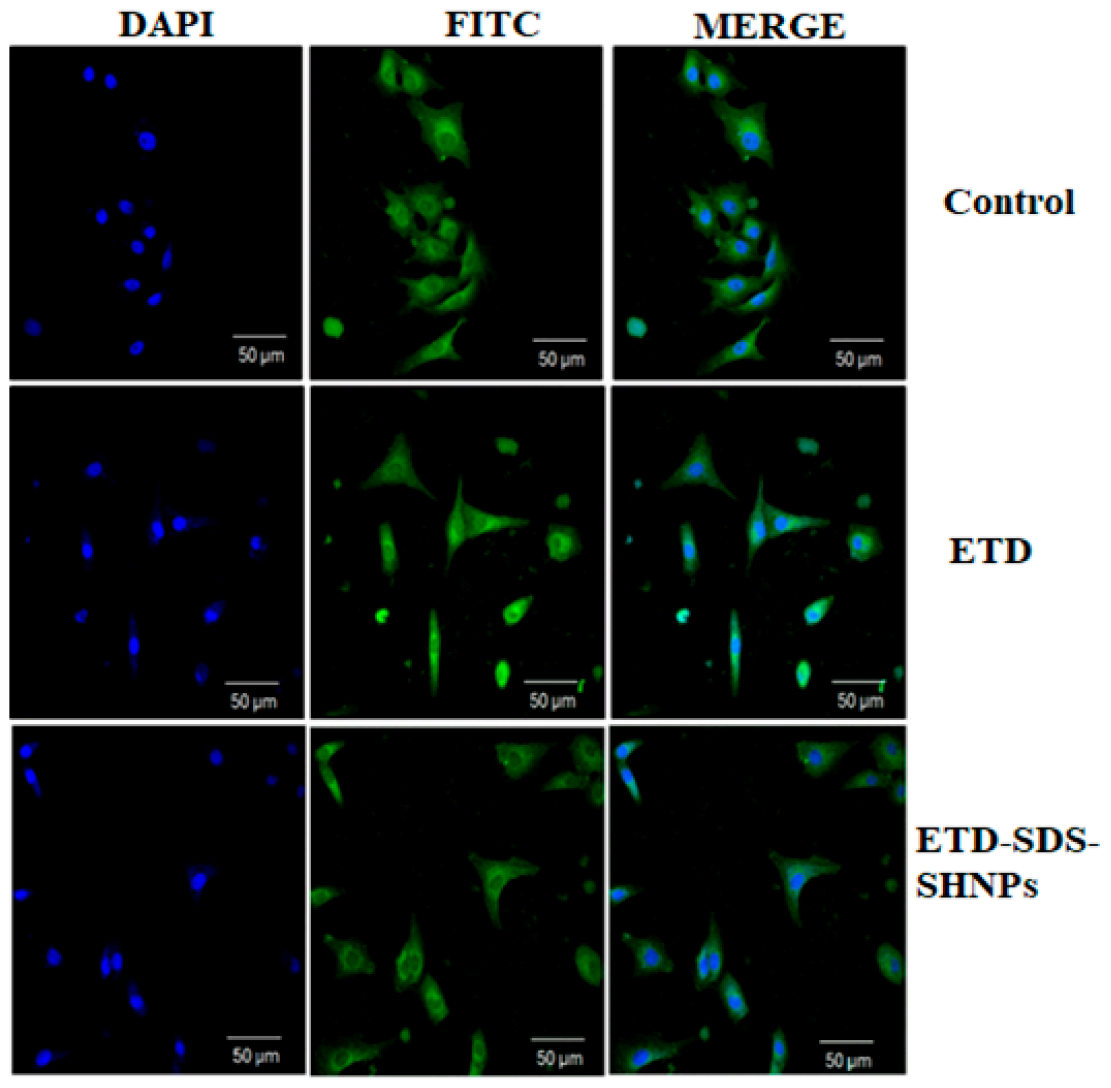
Confocal microscopy images showing cellular uptake of HepG2 cell lines treated with Control, ETD, and ETD-SDS-SHNPs.

The drug ETD and its ETD-SDS-SHNPs formulation are confirmed to be cellularly absorbed by HepG2 cells using confocal microscopy. FITC indicates the presence of the drug (green), while DAPI labels the nuclei (blue). There was no drug uptake in the control group, as evidenced by their blue fluorescence. The cytoplasm of free ETD-treated cells showed measured green fluorescence, indicating limited internalisation. On the other hand, cells treated with ETD-SDS-SHNPs displayed strong, pervasive green fluorescence, suggesting noticeably increased uptake (Figure 11). The small size of the nanoparticle and the presence of sodium deoxycholate and SH, which facilitate membrane penetration, are responsible for this enhanced internalisation. The co-localization of FITC and DAPI signals in the merged images validates cytoplasmic drug accumulation and emphasises the formulation’s superior delivery efficiency [62]. The control group shows a baseline relative intensity of ~15.51, representing natural background fluorescence. Surprisingly, the free drug (ETD) shows a much lower uptake (~2.51), even lower than the control, indicating poor cellular permeability or rapid efflux/metabolism of the unformulated ETD. In contrast, the nanoparticle-based formulation (ETD-SDS-SHNPs) achieves the highest uptake (~32.98), more than doubling the control and outperforming the free drug by a wide margin. This stark contrast highlights the superior delivery efficiency of ETD-SDS-SHNPs in enhancing ETD internalization into HepG2 cells. Overall, the bar graph shows that although free ETD accumulates poorly, its nanoparticle formulation significantly enhances uptake, reinforcing the value of nanocarrier-based strategies for enhanced passive drug delivery. ETD–Shellac nanoparticles exhibited enhanced intracellular uptake in HepG2 cells, enhanced intracellular etodolac delivery, induced S-phase cell-cycle arrest, suppressed inflammatory and proliferative signaling, and provided sustained drug release, thereby improving antitumor efficacy. Similar enhancement in intracellular uptake has been reported for nanosized polymeric nanoparticles, where reduced particle size and improved membrane interaction facilitate efficient cellular internalization [63].

## 4. Conclusions

This study successfully developed and optimized the ETD-SDS-SHNPs formulation, comprising SH and sodium deoxycholate, for the treatment of HCC. The formulation effectively addressed the limitations of free ETD by enhancing its solubility, bioavailability, and therapeutic efficacy. The nanoparticles exhibited favorable physicochemical properties, including nanoscale size, high entrapment efficiency, and sustained drug release, as confirmed by solid-state characterization (FTIR, DSC, XRD, FE-SEM). In vitro studies demonstrated a 2.08-fold increase in cytotoxicity against HepG2 cells, accompanied by S-phase cell cycle arrest and enhanced cellular uptake, indicating enhanced anticancer and antiproliferative activity. In vivo pharmacokinetic analysis revealed improved absorption and prolonged systemic circulation. Overall, ETD-SDS-SHNPs offer an efficient, enhanced passive delivery and safer nanocarrier platform for repurposing ETD in liver cancer therapy, showing significant promise for improving clinical outcomes in HCC treatment.

Study Limitation: A limitation of the present study is that the cytotoxicity of ETD-SDS-SHNPs was evaluated only against HepG2 hepatocellular carcinoma cells. The cytotoxicity of the developed formulation in normal human liver cells was not evaluated due to the unavailability of suitable cell lines and resource limitations. Future studies will focus on evaluating the cytocompatibility, safety, and selectivity of ETD-SDS-SHNPs using normal human hepatocyte cell lines to further establish their biocompatibility.

## Figures and Tables

**Table 1:** Independent and dependent variables used in the BBD for optimization of ETD-SDS-SHNPs.

Independent Variable	Variables Level			Dependent Variable
	Low (−1)	Medium (0)	High (+1)	
X_1_: Amount of shellac (mg)	20	30	40	Particle Size
X_2_: Ultra probe sonication time (min)	15	30	45	Entrapment Efficiency
X_3_: Amount of sodium deoxycholate (mg)	100	200	300	Drug release

**Table 2:** Experimental design run and observed responses obtained from BBD.

Run	Factor 1	Factor 2	Factor 3	Response 1	Response 2	Response 3
	A: Amount of Shellac (mg)	B: Probe Sonication Time (min)	C: Amount of Sodium Deoxycholate (mg)	Particle Size (nm)	Entrapment Efficiency (%)	Drug Release (%)
F1	20	15	200	100 ± 1.8	75 ± 0.82	72 ± 1.73
F2	40	30	300	200 ± 1.8	78 ± 2.16	84 ± 0.82
F3	30	45	300	270 ± 1.63	69 ± 1.63	77 ± 0.63
F4	20	30	100	150 ± 1.24	74 ± 1.73	70 ± 1.73
F5	40	30	100	190 ± 1.94	84 ± 0.82	83 ± 0.82
F6	30	30	200	160 ± 1.63	78 ± 2.16	80 ± 2.16
F7	40	15	200	180 ± 1.82	82 ± 2.45	82 ± 2.45
F8	30	45	100	250 ± 1.82	80 ± 2.16	74 ± 2.16
F9	30	30	200	190 ± 1.83	76 ± 1.73	81 ± 1.73
F10	40	45	200	170 ± 1.24	71 ± 1.41	78 ± 1.41
F11	20	45	200	210 ± 1.82	72 ± 2.16	74 ± 2.16
F12	30	30	200	240 ± 1.63	83 ± 2.16	82 ± 2.16
F13	20	30	300	200 ± 1.82	86 ± 0.82	76 ± 1.82
F14	30	30	200	190 ± 1.63	81 ± 1.63	83 ± 1.41
F15	30	15	300	180 ± 1.94	82 ± 1.41	80 ± 2.62
F16	30	15	100	220 ± 1.82	75 ± 1.63	73 ± 1.63
F17	30	30	200	195 ± 0.6	85 ± 0.8	83 ± 0.82

**Table 3:** Predicted and actual response of checkpoint batches.

Evaluation Parameters	ETD-SDS-SHNPs1			ETD-SDS-SHNPs2		
	Predicted Value	Actual Value	% Error	Predicted Value	Actual Value	% Error
Y_1_: Particle size (nm)	195	192 ± 1.50	1.54%	194	198 ± 1.63	2.02%
Y_2_: Entrapment efficiency (%)	80.6	81 ± 1.42	1.23%	80	78 ± 2.16	2.56%
Y_3_: Drug release (%)	81.8	82 ± 1.25	0.24%	80	79 ± 1.42	1.27%

**Table 4:** In vitro drug release kinetics mathematical models.

Formulation	Zero Order (R^2^)	First Order (R^2^)	Higuchi (R^2^)	Korsmeyer Peppas (R^2^/n)
ETD-SDS-SHNPs	0.0140	0.9605	0.8453	0.9288/0.391

**Table 5:** Results of accelerated stability studies at conditions (40 °C/75% RH).

Time (Days)	Particle Size (nm)	Entrapment Efficiency (%)	Drug Loading (%)	Drug Release (%)
Initial (0)	192.04 ± 1.50	80.6 ± 1.42	20.15 ± 0.84	81.8 ± 1.25
7	193.02 ± 3.26	80.4 ± 1.63	20.05 ± 0.91	81.6 ± 1.23
14	194.05 ± 2.98	80.2 ± 1.41	19.96 ± 0.86	81.4 ± 1.45
30	195.08 ± 2.18	80.1 ± 1.73	19.90 ± 0.88	81.2 ± 1.18

**Table 6:** Pharmacokinetic parameters after oral administration.

Pharmacokinetic Parameters	API (ETD) (Mean ± SD)	ETD-SDS-SHNPs (Mean ± SD)
T_max_ (h)	7 ± 0.03	10 ± 0.08
C_max_ (µg/mL)	7.15 ± 0.14	11.86 ± 0.12
MRT (h)	99.66 ± 1.69	102.96 ± 1.49
AUC_0–t_ (µg.h/mL)	236.09 ± 4.60	351.63 ± 4.59
Relative Bioavailability (%)		148.96%

Abbreviations: C_max_, maximum plasma concentration; T_max_, time to reach maximum plasma concentration; AUC_0–t_, area under the plasma concentration–time curve from time zero to the last measurable concentration; MRT, mean residence time.

## Data Availability

The original contributions presented in this study are included in the article. Further inquiries can be directed to the corresponding author(s).
